# Dietary Glycine and Methyl Donors Remodel Gut Microbiota to Enhance Collagen Synthesis in Sea Cucumber (*Apostichopus japonicus*)

**DOI:** 10.3390/biology14091246

**Published:** 2025-09-11

**Authors:** Dongsheng Chen, Honglin Pei, Yuchen Chen, Anzheng Liu, Tengyu Xing, Hai Zhang, Luo Wang

**Affiliations:** 1 Liaoning Provincial Key Laboratory of Northern Aquatic Germplasm Resources and Genetics and Breeding, Dalian Ocean University, Dalian 116023, China; aa012165758@gmail.com (D.C.); peihonglin2022@163.com (H.P.); 15524566171@163.com (Y.C.); 17866559033@163.com (A.L.); 15554207010@163.com (T.X.); 15242909775@163.com (H.Z.); 2 Key Laboratory of Mariculture & Stock Enhancement in North China’s Sea, Ministry of Agriculture and Rural Affairs, Dalian Ocean University, Dalian 116023, China

**Keywords:** *Apostichopus japonicus*, body wall collagen, gut microbiota, glycine, methyl donors

## Abstract

Collagen content is a primary indicator of quality traits in aquatic animals, with dietary supplementation currently being the main approach to enhance collagen levels. However, the pathways by which food-derived components mediate host collagen synthesis via the gut microbiota remain unclear. This study investigated the regulatory role of gut microbiota in collagen synthesis within the body wall of the sea cucumber (*Apostichopus japonicus*) under dietary supplementation. The results showed that the groups supplemented with 0.60% choline (DJ), 0.50% betaine (TC), and 2.75% glycine (G) significantly increased the collagen content in the sea cucumber body wall by 8.82%, 21.28%, and 22.13%, respectively, compared to the control group (NC). The composition and metabolic function of the sea cucumber gut microbiota were altered by dietary supplementation. The dominant gut microbiota in the supplemented group were *Achromobacter*, *Ferrimonas*, *Shewanella*, and *Haloferula*, which possess capabilities in amino acid metabolism and the decomposition of organic carbon and nitrogen sources. In addition, metabolic pathways such as amino acid metabolism, carbohydrate metabolism, energy metabolism, and nucleotide metabolism were significantly enriched. Glycine and other key collagen precursors exhibited significantly elevated levels in the gut of supplemented sea cucumbers. Research indicates that dietary supplementation with choline, betaine, and glycine modulates the composition and function of the gut microbiota in sea cucumbers. This supplementation also promotes the accumulation of collagen precursors and influences collagen content in the body wall. The objective of this study is to provide a theoretical basis to enhance the quality and efficiency of the sea cucumber aquaculture industry.

## Simple Summary

Choline, betaine, and glycine are dietary supplements that enhance collagen synthesis in sea cucumbers (*Apostichopus japonicus*). In this study, these supplements were added to the feed and tested in a 49-day experiment. The results showed that dietary supplementation significantly increased collagen content in the body wall by up to 22.13%, compared to the control group. This collagen increase occurred with changes in the gut microbiota. Specific bacteria increased, including *Achromobacter*, *Ferrimonas*, and *Shewanella*. These microbial shifts were associated with elevated hydrolyzed amino acids and enhanced glycine levels in the gut, supporting the microbiota’s role as a potential contributor to body wall collagen content. These findings demonstrate that dietary supplements modulate gut bacterial communities to improve collagen production, enhancing quality traits in sea cucumber aquaculture.

## 1. Introduction

Sea cucumber (*Apostichopus japonicus*), which is regarded as a highly nutritious organism and is an economically valuable species with medicinal and food properties, contains abundant collagen that significantly contributes to this value [1,2].

The gut microbiota, recognized as the “second genome” of the host, forms a dynamic symbiotic ecosystem through multifaceted interactions, including metabolic and immune functions. These microbial activities directly regulate host nutrient absorption and biosynthesis pathways. Through dietary fiber fermentation, the gut microbiota generates short-chain fatty acids (SCFAs; e.g., butyrate) that promote protein synthesis in human skeletal muscle. These metabolites suppress histone deacetylases (HDACs), which upregulate insulin-like growth factor-1 (IGF-1) expression [3]. While the specific microbial mechanisms may differ, studies in aquatic animals also demonstrate links between gut microbiota composition and enhanced host protein utilization. In Pacific white shrimp (*Litopenaeus vannamei*), for instance, the gut abundance of *Bacillus* spp. correlates positively with the host’s protein digestion and absorption capacity. Increased abundance of these bacteria significantly enhanced essential amino acid content in the muscle tissue [4]. Increased abundance of beneficial bacteria (e.g., *Lactobacillus* spp.) in the gut tract of Atlantic salmon (*Salmo salar*) enhances total protein content in skeletal muscle tissue [5]. An increased abundance of butyrate-producing bacteria in the gut microbiota of Pacific white shrimp (*Litopenaeus vannamei*) resulted in a 12% elevation in crude protein content and decreased levels of lipid peroxidation products (e.g., malondialdehyde) in muscle tissue [6]. Furthermore, the gut microbiota synthesizes glycine from dietary and endogenous nitrogen sources. This glycine is absorbed by the host, incorporated into procollagen peptide chains during ribosomal translation, and modified by hydroxylase enzymes to form stable collagen structures, thus regulating host collagen synthesis [7,8].

Dietary intake constitutes the predominant regulator of gut microbiota composition and functionality. Specific nutritional components modulate host–microbe interactions by orchestrating microbial growth dynamics and metabolic activity. Research indicates that choline, betaine, and glycine serve as pivotal metabolites in collagen biosynthesis [9]. Dietary supplementation with these compounds enhances quality traits across diverse aquatic species. In Pacific white shrimp (*Litopenaeus vannamei*), dietary choline activates the Adenosine 5‘-monophosphate (AMP)-activated protein kinase (AMPK)/ Sterol Regulatory Element-Binding Protein (SREBP) signaling pathway, enhancing fatty acid β-oxidation and suppressing de novo lipogenesis. This cascade significantly reduces total lipids and triglycerides in the hepatopancreas while attenuating visceral adiposity [10]. In grass carp (*Ctenopharyngodon idella*), dietary choline supplementation enhanced muscle quality traits, characterized by increased proportions of essential amino acids and unsaturated fatty acids, stabilization of muscle pH at near-neutral levels (6.8–7.2), and a 10–15% reduction in shear force [11]. Similar effects were observed in *Oreochromis niloticus* [12] and *Salmo salar* [13], where dietary choline supplementation enhanced muscle protein and lipid content, thereby optimizing texture. By contrast, betaine demonstrated broader efficacy than choline. Under high-fat stress, 20 g/kg betaine reduced hepatic lipid droplet volume and muscle triglyceride levels in *Acanthopagrus schlegelii* via modulation of the Silent Information Regulator 1 (Sirt1)/ Sterol Regulatory Element-Binding Protein-1 (SREBP-1)/ Perixisome Proliferator-Activated Receptor alpha (PPARα) pathway [14]. In gibel carp (*Carassius auratus*), betaine demonstrated dual regulatory effects by concurrently enhancing Very Low Density lipoprotein (VLDL) assembly via the Hepatocyte Nuclear Factor 4 alpha (HNF4α)/ Microsomal Triglyceride Transfer Protein (MTTP) pathway and activating Perixisome Proliferator-Activated Receptor alpha (PPARα)-driven lipolytic catabolism, thus reducing hepatic lipid accumulation while increasing the proportion of unsaturated fatty acids in muscle tissue [15]. Additionally, through its osmoprotectant role, betaine enhanced gut nutrient absorption capacity in Nile tilapia (*Oreochromis niloticus*), increasing muscle crude protein content by 12.5% while reducing lipid content [16]. Glycine exerts multifaceted effects on quality trait improvement in aquatic animals. Dietary glycine supplementation significantly increased collagen content in fish muscle, enhancing textural elasticity and tenderness [17]. In hybrid striped bass (*Morone saxatilis* × *M. chrysops*), dietary glycine (1–2%) significantly elevated muscle creatine concentration and enhanced firmness [18]. Concurrently, in yellow catfish (*Pelteobagrus fulvidraco* × *P. vachelli*), 2–4% glycine supplementation reduced muscle crude fat content (9.55% vs. 12.19% in controls), decreased abdominal lipid deposition, and improved flesh homogeneity [19].

However, the microbial mechanisms through which these dietary supplements mediate collagen synthesis in echinoderms, particularly in sea cucumbers, remain largely unexplored. Therefore, this study aimed to investigate the hypothesis that dietary choline, betaine, and glycine enhance body wall collagen deposition in *A. japonicus* by modulating the composition and metabolic functions of the gut microbiota.

## 2. Materials and Methods

### 2.1. Experimental Materials

Sea cucumbers (5.52 g ± 0.12 g) were acclimatized in a recirculating aquaculture system (T 19.0 °C ± 1.0 °C, salinity 30.0 ± 1.0, DO 6.5 mg/L ± 0.3 mg/L) for 7 days. Throughout acclimation and the subsequent feeding trial, animals were fed every two days with a single ration equivalent to 5% of their initial body weight. Following acclimation, the 480 sea cucumbers were randomly divided into four groups (*n* = 120 per group) to receive the following dietary treatments: NC (control, basal diet), DJ (basal diet supplemented with 0.60% choline), TC (basal diet supplemented with 0.50% betaine), and G (basal diet supplemented with 2.75% glycine). The supplementation levels were selected based on previous pre-experiments and effective doses reported in studies with other aquatic species [20,21]. The feeding trial lasted for 49 days. Culture conditions were identical across all groups and matched those during acclimation.

### 2.2. Sample Processing

At the end of the feeding trial, all sea cucumbers were weighed. The sea cucumbers were anesthetized using a menthol-saturated 10% ethyl alcohol seawater solution diluted to 40% with seawater [22]. The sea cucumber samples were dissected aseptically. Gut contents were collected into sterile 1.5 mL centrifuge tubes, immediately snap-frozen in liquid nitrogen, and stored at −80 °C until DNA extraction. The body wall of sea cucumbers was dissected and sampled for subsequent collagen analysis. For subsequent biochemical and molecular analyses, gut content and body wall samples from 24 individuals per group were randomly pooled to form 3 biological replicates.

### 2.3. Collagen Content Determination

Body wall samples were used for collagen content analysis. Freeze-dried body wall samples were homogenized in 0.1M acetic acid (1:10 *w*/*v*), and collagen content was quantified using a commercial Type I Collagen Sandwich ELISA Kit (Wuhan, China)according to the manufacturer’s instructions. The kit uses specific capture and detection antibodies. Absorbance was measured at 450 nm. A standard curve was generated using the purified type I collagen standard provided in the kit (concentration range: 0–100 ng/mL). Samples were appropriately diluted in the provided assay buffer before analysis.

### 2.4. Hydrolyzed Amino Acid Content Determination

Gut content samples were pooled for hydrolyzed amino acid analysis. Amino acid standards (Sigma-Aldrich AA-S-18 kit, Solarbio, Beijing, China) were used to construct calibration curves with excellent linearity (R^2^ > 0.99). Samples were hydrolyzed with 5 mL 6M HCl at 110 °C for 22 h. After cooling, the hydrolysates were diluted to 10 mL. A 100-μL aliquot was vacuum-dried, derivatized under N_2_ with 50 μL fresh reagent (ethanol:phenylisothiocyanate:water:triethylamine = 7:1:1:1) at 25 °C for 30 min, then diluted with 450 μL mobile phase A (0.1% formic acid) and filtered (0.45 μm). Chromatography was performed on a C18 column (4.6 × 250 mm, 5 μm) at 40 °C with UV detection (254 nm). Mobile phase A: 0.1M sodium acetate-acetonitrile (97:3, pH 6.5); B: acetonitrile–water (80:20) with gradient elution. The amino acid content was calculated as: W = (C − C_0_) × V × N/m (mg/kg). Where C and C_0_ are the sample and blank concentrations (μg/mL), respectively, V is the final volume (mL), N is the dilution factor, and m is the sample mass (g).

### 2.5. High-Throughput Sequencing

Gut tissue samples were used for DNA extraction and subsequent sequencing. Total genomic DNA was extracted from sea cucumber gut contents using the FastDNA Spin Kit for Soil (MP Biomedicals, Santa Ana, CA, USA) according to the manufacturer’s instructions. The V3-V4 hypervariable regions of the bacterial 16S rRNA gene were amplified using the primer pair 515F (5′-GTGCCAGCMGCCGCGGTAA-3′) and 806R (5′-GGACTACHVGGGTWTCTAAT-3′), which is widely used in microbial ecology studies, such as the Earth Microbiome Project [23]. PCR amplification was performed using Phusion^®^ High-Fidelity DNA Polymerase (New England Biolabs, Ipswich, MA, USA) under the following thermal cycling conditions: initial denaturation at 98 °C for 30 s; 32 cycles of denaturation at 98 °C for 10 s, annealing at 54 °C for 30 s, and extension at 72 °C for 45 s; followed by a final extension at 72 °C for 10 min. Sequencing libraries were constructed with the TruSeq^®^ DNA PCR-Free Sample Preparation Kit (Illumina, San Diego, CA, USA), quantified using a Qubit™ 4.0 Fluorometer (Thermo Fisher Scientific, Waltham, MA, USA) and quantitative polymerase chain reaction (qPCR), and assessed for quality. Qualified libraries were subjected to high-throughput sequencing on the NovaSeq 6000 platform (Illumina, San Diego, CA, USA) to generate paired-end reads (2 × 250 bp).

### 2.6. Data Processing

Raw sequencing data were processed using Trimmomatic (v0.35) to trim low-quality bases (Q-score < 20) and remove sequences < 50 bp. Paired-end reads were merged and filtered with FLASH (v1.2.11). Chimeric sequences were identified and removed using UCHIME (v2.4.2). High-quality sequences were clustered into operational taxonomic units (OTUs) at 97% similarity using the UPARSE algorithm (USEARCH v11.0) (de novo clustering). OTUs representing less than 0.005% of the total sequences were discarded to minimize the effect of sequencing errors. Taxonomic classification of representative OTUs was performed using the RDP classifier against the SILVA ribosomal RNA (SILVA) database (http://www.arb-silva.de/, accessed on 28 December 2024). Functional predictions of gut microbiota were conducted with PICRUSt (v2.1.4) based on Kyoto Encyclopedia of Genes and Genomes (KEGG) pathways.

### 2.7. Statistical Analysis

Alpha diversity indices (Chao1, ACE, Shannon, Simpson) were calculated in QIIME (v1.8.0). Beta diversity was assessed using principal coordinate analysis (PCoA) based on weighted UniFrac distances (R software). Data are expressed as mean ± SD. Normality (Kolmogorov–Smirnov test) and homogeneity of variance (Levene’s test) were verified. Intergroup differences were analyzed by one-way ANOVA and LSD post hoc tests (SPSS v22).

## 3. Results

### 3.1. Collagen Content in the Body Wall of Sea Cucumber

**Figure 1:**
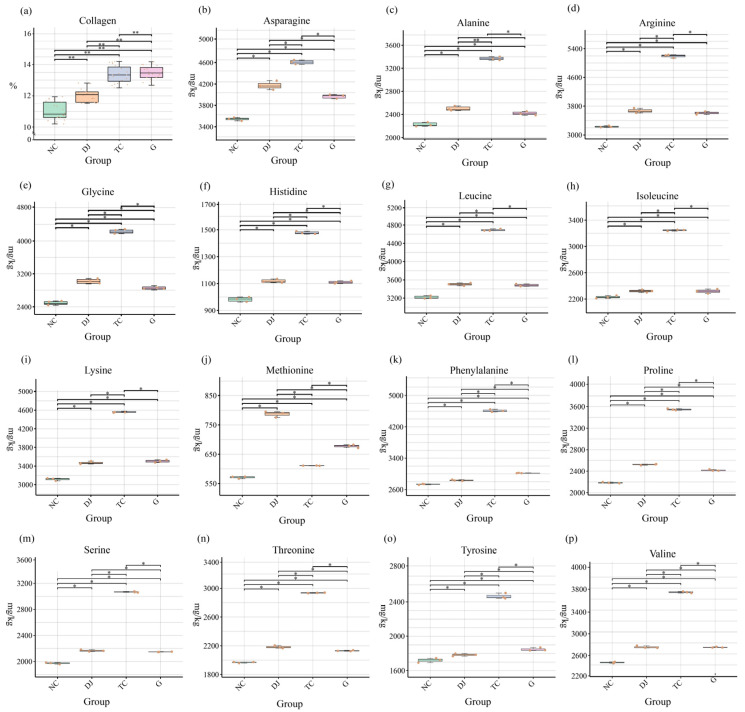
Collagen contents of the body wall and hydrolyzed amino acid contents of the gut in the sea cucumber (*Apostichopus japonicus*). (**a**) Collagen content. (**b**) Asparagine content. (**c**) Alanine content. (**d**) Arginine content. (**e**) Glycine content. (**f**) Histidine content. (**g**) Leucine content. (**h**) Isoleucine content. (**i**) Lysine content. (**j**) Methionine content. (**k**) Phenylalanine content. (**l**) Proline content. (**m**) Serine content. (**n**) Threonine content. (**o**) Tyrosine content. (**p**) Valine content. Note: *: *p* < 0.05; **: *p* < 0.01.

After 49 days of feeding, the final body weight of sea cucumbers showed no significant differences among dietary groups (*p* > 0.05). The collagen content in the sea cucumber body wall was 11.09% ± 0.42% in the NC group. Compared with the NC, dietary supplementation with choline, betaine, and glycine significantly increased collagen levels to 12.11% ± 0.51% (DJ), 13.38% ± 0.49% (TC), and 13.48% ± 0.47% (G), corresponding to relative increases of 8.82%, 21.28%, and 22.13%, respectively (*p* < 0.05) Among the supplements, glycine (G group) was associated with the highest absolute collagen content. (Figure 1a).

### 3.2. Hydrolyzed Amino Acid Content of Sea Cucumber Gut

Dietary supplementation influenced the hydrolyzed amino acid profile in the gut of sea cucumbers (Figure 1b–m), with notable changes in key amino acids. Glycine (Gly) content was highest in the TC group (4218.73 ± 18.77 mg/kg), which was significantly greater than in the NC (2478.74 ± 21.54 mg/kg; *p* < 0.05) and G (2846.81 ± 12.48 mg/kg; *p* < 0.05) groups, representing an increase of 69.89% and 48.18%, respectively. Serine and Arginine(Arg) levels were also significantly elevated in all supplemented groups relative to the NC group (Ser: NC 1972.67 ± 13.56 mg/kg, DJ 2166.14 ± 15.44 mg/kg, TC 3066.91 ± 8.00 mg/kg, G 2147.17 ± 3.43 mg/kg; Arg: NC 3231.21 ± 24.16 mg/kg, DJ 3672.69 ± 67.44 mg/kg, TC 5191.31 ± 43.82 mg/kg, G 3611.48 ± 43.91 mg/kg; *p* < 0.05). Methionine(Met) was notably higher in the DJ group (786.81 ± 9.95 mg/kg) compared to both the NC (571.26 ± 4.15 mg/kg; *p* < 0.01) and G (677.37 ± 5.34 mg/kg; *p* < 0.05) groups.

### 3.3. Structural and Functional Characteristics of Gut Bacterial Community

#### 3.3.1. Analysis of High-Throughput Sequencing Results

Original sequenced reads obtained from 32 samples of sea cucumber treated with different feed additives ranged from 120,499 to 137,374. After sequence optimization, rehybridization, and filtration of short-length fragments, low-quality bases, and chimeric artifacts, high-quality valid sequences ranging from 116,619 to 132,593 were obtained. The proportion of valid sequences exceeded 96.75% with a coverage rate of 99.56%, confirming that the sequencing results accurately reflected the biological characteristics of the samples.

#### 3.3.2. Diversity Analysis of Bacterial Community

**Figure 2:**
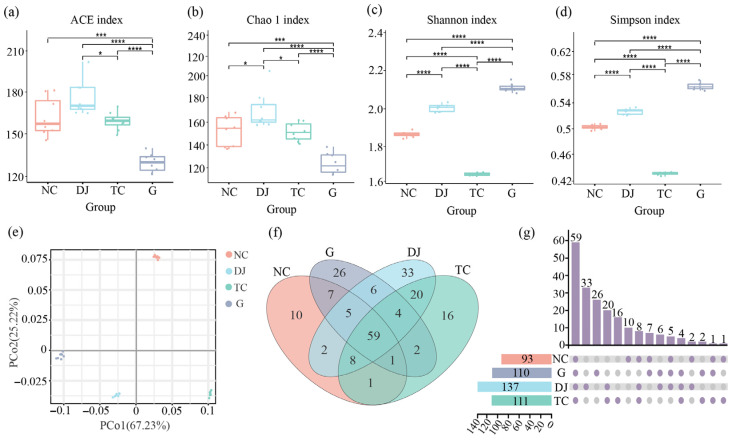
Diversity of gut bacterial community in sea cucumber (*Apostichopus japonicus*). (**a**) ACE index. (**b**) Chao 1 index. (**c**) Shannon index. (**d**) Simpson index. (**e**) PCoA score plots. (**f**) Venn diagram. (**g**) Upset diagram. Note: *: *p* < 0.05; ***: *p* < 0.001; ****: *p* < 0.0001.

Diversity of the gut bacterial community showed significant differences among dietary supplement groups (Figure 2a–d). Compared with the NC group (Chao1: 154.38 ± 12.99; ACE: 162.38 ± 13.87; Shannon: 1.86 ± 0.02; Simpson: 0.50 ± 0.00), the DJ group (Chao1: 170.52 ± 16.27; ACE: 173.01 ± 11.60; Shannon: 2.00 ± 0.02; Simpson: 0.53 ± 0.00) showed significantly increased bacterial community richness and diversity. Specifically, the Chao1 and ACE indices increased by 10.45% and 6.55%, respectively, while the Shannon and Simpson indices increased by 7.53% and 6.00% (*p* < 0.05). In contrast, the TC group (Chao1: 151.80 ± 7.52; ACE: 159.13 ± 5.58; Shannon: 1.66 ± 0.01; Simpson: 0.43 ± 0.00) showed significantly reduced bacterial community richness and diversity compared to NC. Specifically, the Chao1 and ACE indices decreased by 1.67% and 2.00%, respectively, while the Shannon and Simpson indices declined by 10.75% and 14.00% (*p* < 0.05). The G group (Chao1: 125.14 ± 8.56; ACE: 128.20 ± 6.27; Shannon: 2.10 ± 0.03; Simpson: 0.56 ± 0.01) exhibited elevated diversity but reduced richness relative to NC: the Shannon and Simpson indices increased by 12.90% and 12.00%, respectively, whereas the Chao1 and ACE indices decreased by 18.94% and 21.06% (*p* < 0.05).

The first principal coordinate (PCoA1) explained 67.23% of the total variation, and the second principal coordinate (PCoA2) explained 25.22%, totaling a total contribution of 92.45%. Samples from different groups were dispersed in different areas of the plot, indicating a significant difference in the bacterial community structure between groups (*p* < 0.05). Samples within the same group were mostly clustered together, indicating strong biological reproducibility. There was significant variability (*p* < 0.05) in the structure of the bacterial community between groups of samples from the intestine of sea cucumber after different dietary supplements (Figure 2e).

#### 3.3.3. Structural Characteristics of Bacterial Community

**Figure 3:**
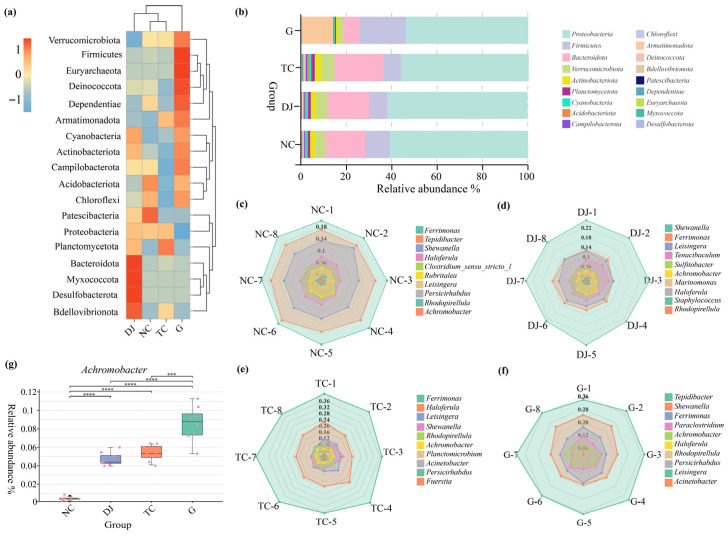
Structural characteristics of gut bacterial community in the sea cucumber (*Apostichopus japonicus*). (**a**) Heat map of bacterial community at phylum levels. (**b**) Structural characteristics of bacterial community at the genus level. (**c**) Relative abundance of the top 10 bacterial genera in the NC group. (**d**) Relative abundance of the top 10 bacterial genera in the DJ group. (**e**) Relative abundance of the top 10 bacterial genera in the TC group. (**f**) Relative abundance of the top 10 bacterial genera in the G group. (**g**) Comparative analysis of the relative abundance of *Achromobacter* in experimental groups. Note: ***: *p* < 0.001; ****: *p* < 0.0001.

The structure of the gut microbiota was reshaped by dietary supplementation (Figure 3a,b). While Proteobacteria and Firmicutes remained the dominant phyla across all groups, the predominant genera shifted. In the NC group, the dominant genera were *Ferrimonas* (20.80%), *Tepidibacter* (17.46%), and *Shewanella* (13.03%). In the supplemented groups, the communities were dominated by *Achromobacter*, *Ferrimonas*, *Shewanella*, and *Haloferula*, but with distinct variations: Shewanella was most abundant in DJ (24.55%), *Ferrimonas* in TC (40.12%), and *Tepidibacter* in G (37.64%) (Figure 3c–f). The relative abundance of *Achromobacter* was higher in all supplemented groups compared to the NC group. The most substantial increase was observed in the G group, where it reached 8.47% (Figure 3g).

#### 3.3.4. Functional Characteristics of Bacterial Community

**Figure 4:**
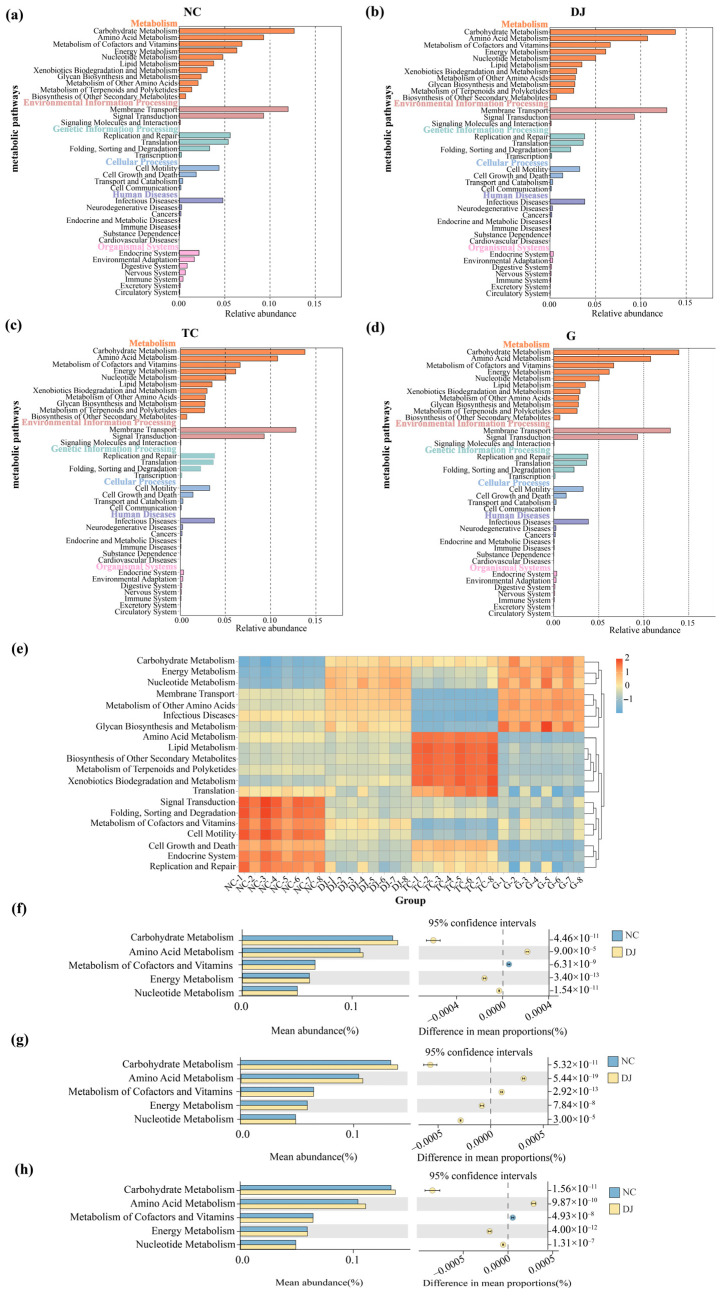
Functional characteristics of the gut bacterial communities in sea cucumber (*Apostichopus japonicus*). (**a**) Differences in the relative abundance of KEGG pathways in the NC group. (**b**) Differences in the relative abundance of KEGG pathways in the DJ group. (**c**) Differences in the relative abundance of KEGG pathways in the TC group. (**d**) Differences in the relative abundance of KEGG pathways in the G group. (**e**) Heat map of significantly enriched KEGG pathways of the gut bacterial communities. (**f**) *t*-test comparison of the top 5 metabolic pathways between the NC and DJ groups. (**g**) *t*-test comparison of the top 5 metabolic pathways between the NC and TC groups. (**h**) *t*-test comparison of the top 5 metabolic pathways between the NC and G groups.

PICRUSt2 analysis, based on the KEGG database, was employed to predict the functional potential of the gut bacterial communities across the dietary groups. A total of 36 level 2 and 170 level 3 KEGG pathways were annotated. Among these, pathways categorized under ‘Metabolism’ constituted the most predominant functional category (Figure 4e). To further investigate the metabolic shifts, we focused on the top five most enriched pathways within the ‘Metabolism’ category: amino acid metabolism, carbohydrate metabolism, metabolism of cofactors and vitamins, energy metabolism, and nucleotide metabolism. Comparative analysis revealed a consistent pattern of functional upregulation in the supplemented groups: Compared to the NC group, the predicted abundances of genes related to amino acid metabolism and carbohydrate metabolism were consistently elevated in all supplemented groups (DJ, TC, and G) (Figure 4f–h).

### 3.4. Correlation Analysis Between Gut Bacterial Community and Body Wall Collagen in Sea Cucumber

**Figure 5:**
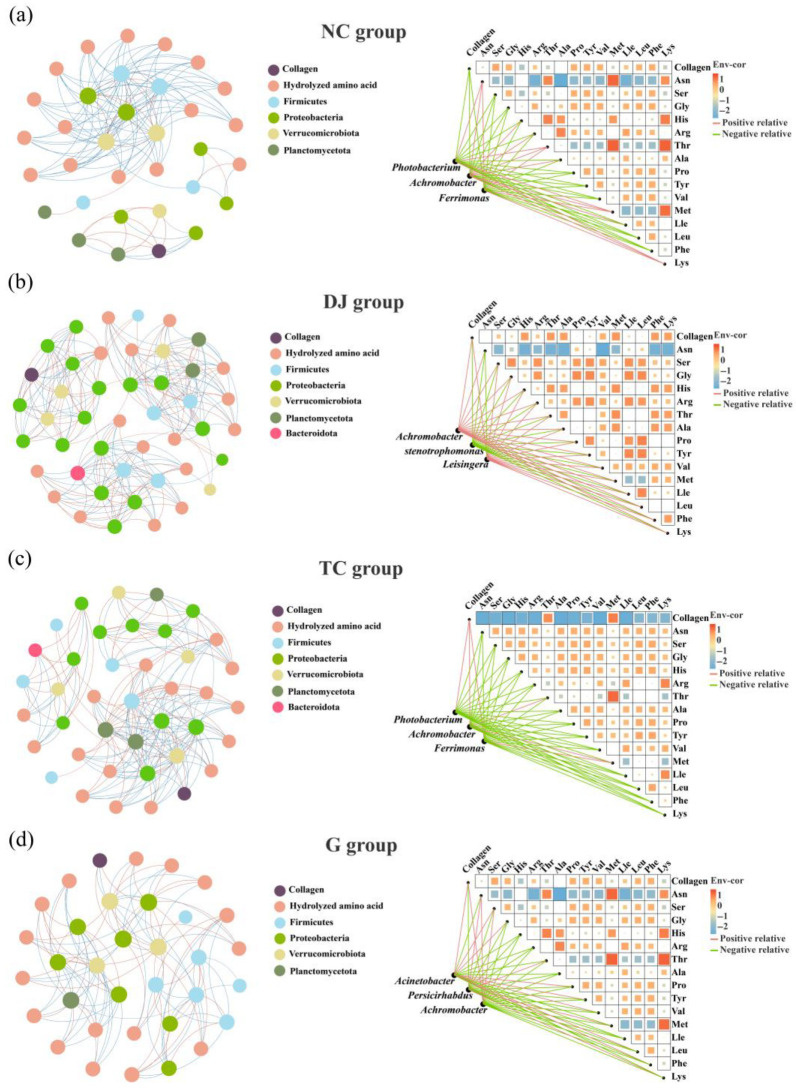
Network and heat map of significant correlations among gut bacterial communities, gut hydrolyzed amino acids, and body wall collagen in sea cucumber (*Apostichopus japonicus*).

Co-occurrence network analysis revealed distinct topological patterns in the gut bacterial communities among dietary treatment groups (Table 1, Figure 5). The network complexity increased in the supplemented groups: the DJ group contained 45 nodes and 239 edges, compared to 33 nodes and 129 edges in the NC group. The core taxa also shifted from *Ferrimonas*, *Tepidibacter*, and *Haloferula* in the NC group to genera such as *Achromobacter*, *Stenotrophomonas*, and *Staphylococcus* in the DJ group. In the TC and G groups, although node counts were 40 and 34, respectively, both were characterized by a high abundance and centrality of *Achromobacter*, which consistently emerged as a keystone genus across all supplemented groups. Notably, *Achromobacter* showed a strong positive correlation with body wall collagen content (Figure 5), suggesting its potential role in enhancing collagen deposition.

## 4. Discussion

### 4.1. Dietary Supplementation Modulates the Gut Bacterial Community

The gut microbiota is modulated by dietary input [24,25,26,27]. In this study, dietary supplementation with choline, betaine, and glycine modulated the gut microbiota structure of sea cucumbers. Supplemented groups showed significant enrichment of *Achromobacter*, while distinct dominant genera emerged in specific treatment groups: *Shewanella* in DJ, *Ferrimonas* in TC, and *Tepidibacter* in G. These heterotrophic genera specialize in degrading complex organic carbon sources.

*Achromobacter*, a genus of Gram-negative bacilli, represents ubiquitous inhabitants of aquatic and soil ecosystems. Members of this genus possess versatile metabolic capabilities, including pathways for amino acid metabolism, carbohydrate catabolism, energy generation, and secondary metabolite biosynthesis. Notably, they exhibit robust degradative capacities for xenobiotic compounds [28]. The significant increase in *Achromobacter* abundance observed in the DJ, TC, and G groups likely reflects an individual promotional effect of choline, betaine, and glycine supplementation, respectively. These compounds act as methyl donors that enhance bile acid conjugation [27]. It is plausible that these metabolic shifts modulate the gut environment, which may, in turn, contribute to the observed proliferation of *Achromobacter*, a genus known for its versatile metabolic capabilities. Biochemical studies confirm that choline-derived betaine participates in one-carbon metabolism by donating methyl groups to homocysteine via betaine-homocysteine methyltransferase (BHMT), generating methionine (Met), while glycine contributes methylene units through the glycine cleavage system [29,30]. These compounds activate the host-derived farnesoid X receptor (FXR), upregulating cholesterol 7α-hydroxylase (CYP7A1) to promote cholesterol conversion into bile acids, thereby modulating intestinal microbiota [31].

*Achromobacter* gains a competitive advantage by efficiently acquiring methyl donors, either through direct uptake of Met via specific transporters (e.g., MetQ) or through endogenous regeneration of Met via methionine synthases (MetE/MetH) [32]. This metabolic flexibility may enhance glycine availability, a key precursor for collagen synthesis, thereby indirectly supporting host collagen production. Furthermore, *Achromobacter* transforms primary bile acids (e.g., cholic acid) into deoxycholic acid (DCA) via bacterial 7α-dehydroxylation, whereby DCA suppresses Gram-positive bacterial proliferation, thereby reducing resource competition [28].

*Shewanella* is a genus of Gram-negative, rod-shaped bacteria that commonly exhibit a single polar flagellum and high motility [33]. Members of this genus are oxidase- and catalase-positive, facultatively anaerobic, and widely distributed in diverse environments, including marine ecosystems, aquatic animal guts, and sediments [34]. A potential mechanism underlying *Shewanella* enrichment in the DJ group may involve its unique metabolic capabilities for utilizing choline-derived metabolites. Specific gut microbiota (e.g., Proteobacteria and Firmicutes) convert choline to trimethylamine (TMA) via choline TMA-lyase (CutC/D) [35]. TMA enters the liver via the portal vein, where it is oxidized to trimethylamine-N-oxide (TMAO) by flavin-containing monooxygenase 3 (FMO3) [25]. *Shewanella* encodes the TMAO reductase system (e.g., TorA/TorC), which reduces TMAO back to TMA under anaerobic conditions while generating ATP via the electron transport chain, thereby driving its energy metabolism and proliferation [36,37].

*Ferrimonas* are Gram-negative, facultatively anaerobic rods exhibiting oxidase and catalase activities, with optimal growth under mesophilic conditions (25–28 °C), neutral to slightly alkaline pH (7.0–9.0), and moderate salinity (0.5–8% NaCl, optimal 3%) [38,39]. Betaine accumulates intracellularly to counterbalance osmotic stress in the gut environment, enhancing microbial survival under hyperosmotic conditions and promoting facultative anaerobe proliferation [40,41]. This osmotic adaptation mechanism likely facilitates *Ferrimonas* colonization in the gut.

*Tepidibacter* comprises Gram-positive, spore-forming anaerobic or aerotolerant fermentative bacteria prevalent in extreme environments and animal guts [42,43]. Certain species exhibit unique metabolic capabilities, such as utilizing amino acids as carbon sources. While most gut microbes show limited glycine utilization, *Tepidibacter mesophilus* specifically metabolizes mixed DL-alanine and L-glycine as sole carbon substrates, a trait linked to genomic encoding of the glycine cleavage system (GCS) and other amino acid deaminases [44]. Glycine degradation generates acetate and ethanol, acidifying the gut lumen [45], thereby inhibiting pH-sensitive microbiota while creating favorable niches for acid-tolerant or spore-forming *Tepidibacter.*

The observed gradient in collagen enhancement (G > TC > DJ) despite the convergent enrichment of *Achromobacter* raises an important point about nutrient source. The superior performance of direct glycine supplementation could be attributed to its high bioavailability, allowing for efficient direct absorption and utilization by the host. In contrast, methyl donors like choline and betaine participate in numerous competing metabolic pathways in the host (e.g., phospholipid synthesis, antioxidant responses), which may divert a portion of these compounds away from supporting microbial glycine synthesis [29], thus resulting in a relatively smaller net effect on collagen deposition.

### 4.2. Gut Microbiota Modulates Hydrolyzed Amino Acid Synthesis

Hydrolyzed amino acids generated via protein hydrolysis comprise amino acids originating from undigested dietary proteins, endogenous host proteins, and microbial metabolites [46]. The gut microbiota participates in intricate metabolic exchanges involving these hydrolyzed amino acids. On one hand, gut microbes secrete proteases that degrade both dietary- and host-derived proteins into oligopeptides and free amino acids. These substrates undergo microbial deamination and decarboxylation, producing metabolic end-products such as short-chain fatty acids (SCFAs), ammonia, and hydrogen sulfide [47]. These compounds subsequently support microbial proliferation and modulate host physiological functions via enhanced nutrient absorption and signaling pathways [48]. On the other hand, the microbiota utilizes hydrolyzed amino acids for de novo microbial protein biosynthesis. Through transamination reactions, incompletely digested proteins or peptides are converted into bioavailable amino acids, including essential amino acids [49].

The significant increase in gut hydrolyzed amino acid content observed in supplemented groups may stem from the combined effects of upregulated microbial amino acid metabolism and *Achromobacter*-specific metabolic activities. Enhanced metabolic activity of the microbial community, combined with synergistic input from exogenous protein-derived nitrogen sources, likely induced a metabolic shift that suppressed endogenous biosynthetic pathways while promoting degradation of dietary and host-derived proteins. Concurrently, *Achromobacter* amplified glycine and other critical amino acid supplies through targeted metabolic processes, including choline oxidation and collagenase-specific hydrolysis. These combined mechanisms may have collectively contributed to the elevated levels of hydrolyzed amino acids, particularly glycine, in the gut of sea cucumbers.

Bacterial amino acid metabolism encompasses the synthesis, degradation, and interconversion of all 20 standard amino acids, including glycine, serine, threonine, alanine, aspartate, and glutamate. This process involves the activity of various secreted proteases (e.g., serine proteases, metalloproteases) and peptidases (e.g., aminopeptidases, carboxypeptidases) [50,51]. The upregulated expression of microbial amino acid metabolism promoted the release of intermediate metabolites such as glycine and glutamate. Concurrently, enhanced protease and peptidase expression facilitated the hydrolysis of dietary and endogenous proteins into short peptides and free amino acids, thereby elevating gut hydrolyzed amino acid content. Furthermore, the supplementation of choline, betaine, and glycine provided exogenous nitrogen sources for amino acid synthesis. This suppressed bacterial endogenous amino acid synthesis while increasing the release of hydrolyzed amino acids. Research indicates that the enzymatic activities of bacterial 1-aminocyclopropane-1-carboxylate (ACC) deaminase and exogenous hydrolases are closely associated with microbial growth and metabolic capacity [52]. Under specific conditions, exogenous hydrolases can significantly increase amino acid release, thereby influencing microbial metabolic efficiency [53]. When external nitrogen sources are sufficient, bacteria preferentially decompose exogenous amino acids rather than synthesize endogenous ones, leading to net accumulation of hydrolysis products [53]. Consequently, the upregulation of amino acid metabolic pathways drives elevated gut hydrolyzed amino acid levels through enhanced enzyme activity and optimized nitrogen source allocation.

*Achromobacter* possesses a unique oxidative pathway to metabolize choline and betaine into dimethylglycine, independent of the transmethylation process [54]. This bacterium preferentially utilizes choline as a carbon and nitrogen source but lacks metabolic capacity for methylaminoethanol or trimethylamine. The pathway employs Flavin Adenine Dinucleotide (FAD) as a cofactor, relying on choline oxidase to catalyze the oxidation of choline to betaine, which is further oxidized to formaldehyde and dimethylglycine [55]. Dimethylglycine serves as a direct precursor for glycine biosynthesis [56].

In the presence of collagen, *Achromobacter* activates the transcription of the collagenase gene *colH* via the PhoP-PhoQ two-component regulatory system and σ factors, which perceive substrate signals through membrane receptors and transporter-mediated signal transduction [57]. The collagenase EC 3.4.24.8, secreted via the Selenocysteine or Tat(YGRKKRRQRRR) pathways, specifically cleaves the X-Gly bond within Gly-X-Y repeating motifs of collagen, such as Gly-Ala-Hyp-Gly or Gly-Pro-Hyp-Gly, overlapping partially with eukaryotic collagenase cleavage sites at interbands 33–34 and 41–42 [58]. This enzymatic hydrolysis generates glycine, proline, and hydroxyproline, which serve as carbon/nitrogen sources and precursors for host collagen synthesis.

An intriguing question arising from our results pertains to the relative contribution of the proposed pathways (choline oxidation vs. collagenase activity) through which *Achromobacter* may supply glycine. While both pathways are plausible, the superior efficacy of direct glycine supplementation (G group) in enhancing body wall collagen, compared to methyl donors (DJ and TC groups), might suggest a higher bioavailability of pre-formed dietary glycine. Conversely, the highest gut glycine content was observed in the TC (betaine) group, hinting at a potent stimulation of de novo microbial glycine production from this methyl donor. This suggests a complex interaction where different supplements may preferentially activate distinct microbial pathways to influence the host glycine pool, a hypothesis that warrants further investigation.

### 4.3. Hydrolyzed Glycine Promotes Collagen Synthesis

Glycine is a primary component of collagen; the significantly elevated glycine levels we observed in the gut could potentially have supported the enhanced collagen synthesis found in the body wall. Studies demonstrate that glycine availability is a critical rate-limiting factor for collagen synthesis. In vitro and cellular models show that increasing extracellular glycine concentration significantly enhances procollagen production, while impaired glycine transport directly suppresses collagen output in producing cells [59]. This underscores the fundamental importance of glycine abundance. Biochemical and cellular studies demonstrate that glycine is actively transported into collagen-producing cells primarily via specific transporters such as GlyT1. Following cellular uptake, glycine is directly incorporated into the Gly-X-Y repeats of nascent procollagen chains during ribosomal translation [60]. Glycine directly supports the post-translational modification of procollagen chains and subsequent collagen fibril assembly. Furthermore, glycine metabolism generates one-carbon units essential for hydroxylase cofactor synthesis, thereby enabling hydroxylation of proline/lysine residues critical for collagen stabilization [61].

In this study, dietary supplementation with choline, betaine, and glycine significantly increased glycine concentrations in the sea cucumber gut. Concomitantly, supplemented groups exhibited elevated collagen content in the body wall. This observed link between elevated gut glycine levels and increased body wall collagen content in sea cucumber aligns with evidence demonstrating that dietary glycine supplementation (0.5–2% feed inclusion) enhances collagen synthesis across diverse species, evidenced by increased collagen content in swine [62], poultry [63,64], *Litopenaeus vannamei* [65], salmon [66], and *Scophthalmus maximus* [67].

## 5. Conclusions

In conclusion, this study demonstrates that dietary supplementation with choline, betaine, or glycine significantly enhances body wall collagen content in *A. japonicus*. This enhancement may be mediated through a restructuring of the gut microbiota, notably by enriching *Achromobacter*, which is associated with elevated levels of hydrolyzed amino acids, particularly glycine, in the gut. These findings provide a novel microbial perspective for improving collagen deposition and overall quality in sea cucumber aquaculture through dietary intervention. Future studies employing germ-free models or fecal microbiota transplantation (FMT) experiments are needed to definitively establish the causal role of the identified microbial community in mediating collagen synthesis.

## Figures and Tables

**Table 1:** Topological indices of the symbiotic network of intestinal bacterial community in sea cucumber (*Apostichopus japonicus*).

Network Metrics/Group	DJ	G	NC	TC
Number of nodes	45	34	33	40
Number of edges	239	113	129	190
Number of positive	104	48	29	75
Number of negative	134	65	100	115
Graph Density	0.24	0.201	0.244	0.244
Network diameter	4	4	5	5
Average clustering coefficient	0.844	0.844	0.847	0.871
Average degree	10.578	6.647	7.818	9.5
Modularity	0.642	0.652	0.293	0.483
Number of nodes	45	34	33	40

## Data Availability

The original contributions presented in the study are included in the article. If there is a request, further inquiries can be directed to the corresponding author.
